# Birth weight by gestational age and congenital malformations in Northern Ethiopia

**DOI:** 10.1186/s12884-015-0507-2

**Published:** 2015-03-29

**Authors:** Hayelom K Mekonen, Balkachew Nigatu, Wouter H Lamers

**Affiliations:** 1 Department of Anatomy, College of Health Sciences, Mekelle University, Mekelle, Ethiopia; 2 Department of Gynaecology and Obstetrics, College of Health Sciences, Mekelle University, Mekelle, Ethiopia; 3 Department of Anatomy & Embryology, Faculty of Health, Medicine, and Life Sciences, Maastricht University Medical Center, Maastricht, The Netherlands

**Keywords:** (Very) low birth weight, Sex difference, Parity, CNS malformations, Northern Ethiopia

## Abstract

**Background:**

Studies on birth weight and congenital anomalies in sub-Saharan regions are scarce.

**Methods:**

Data on child variables (gestational age, birth weight, sex, and congenital malformations) and maternal variables (gravidity, parity, antenatal care, previous abortions, maternal illness, age, medication, and malformation history) were collected for all neonates delivered at Ayder referral and Mekelle hospitals (Northern Ehthiopia) in a prospective study between 01-12-2011 and 01-05-2012.

**Results:**

The total number of deliveries was 1516. More female (54%) than male neonates were born. Birth weights were 700-1,000 grams between 26 and 36 weeks of pregnancy and then increased linearly to 3,500-4,000 grams at 40 weeks. Thirty-five and 54% of neonates were very-low and low birth weight, respectively, without sex difference. Very-low birth-weight prevalence was not affected by parity. Male and female neonates from parity-2 and parity-2-4 mothers, respectively, were least frequently under weight. Sixty percent of newborns to parity -3 mothers weighed less than 2,500 grams, without sex difference. The percentage male neonates dropped from ~50% in parity-1-3 mothers to ~20% in parity-6 mothers. Diagnosed congenital malformations (~2%) were 2-fold more frequent in boys than girls. The commonest malformations were in the central nervous system (CNS; ~1.5% of newborns). Parity, low birth weight, gestational age less than 35 weeks, male sex, and lack of antenatal care were the most significant risk factors for congenital anomalies.

**Conclusion:**

The high prevalence of neonates with low birth weight and CNS anomalies in Northern Ethiopia was very high. The findings may reflect the harsh conditions in the past 2 decades and suggest environmental and/or nutritional causes. Male sex and parity affected the outcome of pregnancy negatively.

## Background

Birth weight is a technically simple parameter to monitor prenatal health in a population. Establishing the prevalence of low birth weight (LBW) is particularly important, since perinatal morbidity and mortality are more frequent in LBW than in normal infants and has become the second cause of death in this period, after premature birth [1]. It has been shown that a sub-optimal weight at birth may impair neurological function and can cause chronic disease, such as hypertension in the perinatal period, during infancy, and even in adulthood [2]. Prenatal growth retardation, premature birth, and congenital malformations appear the most important factors that determine low birth weight [3]. Furthermore, socio-economic factors like habitat, education, birth order, age, and religion also affect birth weight [4]. Moreover, women born with LBW are more likely to give birth to infants with LBW, contributing to the trans-generational cycle of malnutrition and poverty [5]. In line with these arguments, most cases of LBW in Africa are attributed to intrauterine growth retardation (IUGR) rather than to preterm delivery [6]. From all live births worldwide, 16% are LBW, 20% of which occur in the low-income sub-Saharan African countries [7]. In Gambia, for example, ~20% of infants were estimated to weigh <2,500 g at birth [8]. Although numerous factors interact with and affect fetal development, maternal malnutrition, particularly micronutrient deficiences is assumed to be a major determinant of IUGR [9].

The number and especially the type of congenital malformations in newborns can also reflect prenatal health in a population. The global prevalence of all congenital malformations is 2-3% [10], while the reported incidence of malformations of the various systems of the body ranges from 1 to 5% [11]. Congenital anomalies are a significant challenge to public health, since they are responsible for ~7% of all under-five deaths [12]. As infectious diseases and malnutrition are being addressed in developing countries, congenital malformations will probably assume a greater relative importance as a cause of mortality and morbidity among infants and children [13].

Although underreporting, deficiencies in diagnostic capabilities, and poor follow-up at birth may affect the report of congenital malformations [14], the actual prevalence of congenital anomalies in Africa probably differs from that in the developed world due to differences in exposure to e.g. maternal infections and malnutrition. However, few reports exist and the available percentages are likely underestimates because they often rely on verbal autopsy studies [15], while differences in study design, methods of ascertainment, and the apparent observer-dependent incidence of congenital malformations make them difficult to compare [14].

No prospective study on birth weight and the incidence of congenital anomalies is, to our knowledge, available for Northern Ethiopia. We, therefore, started such a study to obtain a first impression of neonatal health at birth, the frequency of congenital anomalies, and potential risk factors in the Ayder refferral and Mekelle zonal hospitals. Ayder referral hospital is a teaching hospital under Mekelle University and administered by the Ministry of Education. The hospital has 457 beds and provides full medical services. The hospital labor ward has 6 waiting beds, 3 couches and currently sees 1,000-1,100 deliveries per year. Mekelle zonal hospital is the oldest hospital in Mekelle city and administered by the Ministry of Health. It has 240 beds and a labor ward with 2 waiting beds, 3 couches and currently around 2,400 deliveries per year. Though the estimated number of deliveries in the town and surrounding district Enderta in the study period was ~6,000, only 1,516 (~25%) deliveries were attended in both hospitals. In general, ~6% deliveries in Ethiopia are attended in health institutions [16]. The low number of deliveries in the study hospitals is due to a higher number of home deliveries. The aim of the study was to investigate the distribution of birth weights in the sample population and the incidence and associated factors of congenital malformations at birth in both hospitals between 01-12-2011 and 31-05-2012.

## Methods

This prospective cohort study was conducted between 01-12-2011 and 31-05-2012 and included all deliveries. Data on child variables (gestational age, birth weight, sex, and congenital malformations) and maternal variables (gravidity, parity, antenatal care, previous abortions, maternal illness, age, medication and malformation history) were collected at Ayder referral and Mekelle hospitals. Birth weight, gestational age, gravidity, parity and maternal age were considered as continuous variables, while sex, antenatal care, previous abortions, maternal illness, medication, malformation history were categorical. Births outside the two hospitals were excluded. The births were attended by midwives and midlevel obstetric professionals (trained in emergency surgery and obstetrics). Birth weight was measured using baby weighing scale pan type in which the weight was estimated to the nearest 100grams. Neonates weighing <1,500 and <2,500 grams were considered very low birth weight (VLBW) and low birth weight (LBW), respectively. Assesment of gestational age was deduced from the last reported menstrual period. Ultrasound examinations were not carried out routinely, because the equipment was not available in the antenatal care centers. All relevant information about birth weight and the presence of gross congenital defects detected within 24 hours after delivery was collected. The data was entered into a predesigned data collection format. The distribution of gestational age, parity, birth weight and sex was visualized as frequencies or proportions. The association of malformations with child and maternal variables was analyzed with classical logistic regression including the confounders, using Stata version 13. A P value of <0.05 was considered significant and <0.10 as indicating a trend. The ethical clearance for the study was obtained from Mekelle University-College of Health Sciences, Health Research Ethics Review Committee (HRERC) within Research and Community Service Team. Verbal informed consent was obtained from the mothers, as most of them were illiterate, prior to the beginning of the interviews.

## Results

### Birth weight characteristics of the cohort

**Figure 1:**
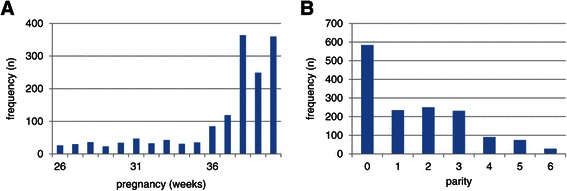
**Frequency distribution of newborns.** Distribution of gestational age **(panel A)** and parity **(panel B)** in the cohort.

**Figure 2:**
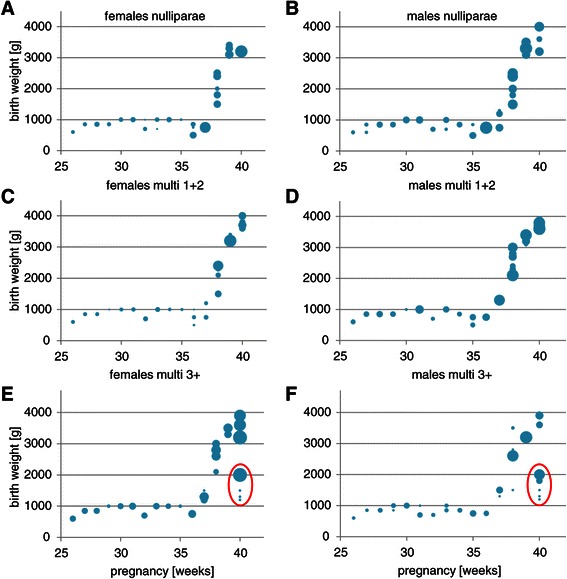
**Attained birth weight as a function of gestational age in female (panels A, C and E) and male (panels B, D and F) neonates.** The upper row (panels A and B) shows data for female and male offspring, respectively of nulliparous mothers, the middle row (panels C and D) for female and male offspring, resp. of mothers with 1 or 2 previous children and the bottom row (panels E and F) for female and male offspring, resp. of multiparous mothers with more than 3 children. The red circle contains neonates with a birth weight <2,000 gram born at 40 weeks. The size of the bubbles represents the number of infants in the group. The smallest bubble represents a single neonate and the largest 51 neonates.

**Figure 3:**
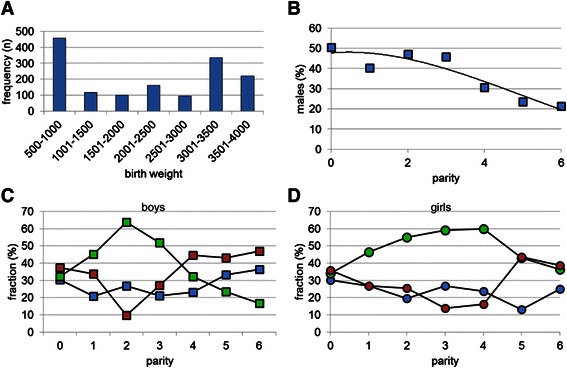
**Characteristics of newborns.** Distribution of birth weights in the cohort (**panel A**), the percentage of boys among offspring as a function of parity (**panel B**; R^2^ = 0.85 with 3^rd^ order polynomial), and the percentage offspring with a birth weight <1,500 gram (blue symbols), between 1,500 and 2,500 gram (red symbols), or >2,500 gram (green symbols) (**panel C** for male and **panel D** for female infants).

The total number of deliveries in the two hospitals during the 6-months study period was 1,516. Of these, 1484 had no diagnosed malformation (Table 1). More female (55%) than male neonates were born (P < 0.001; Table 1). Twenty-eight and 13% of the babies were born before 37 and 32 weeks, respectively (Figure 1A). Sixty percent of neonates were born from the multiparous mothers (Figure 1B). Figure 2 shows that these newborns had birth weights of 700-1,000 grams if born between 26 and 36 weeks of pregnancy, followed by a strong, linear increase in birth weight of ~800 grams per week during the last 4 gestational weeks. In total, 35% and 54% of the neonates weighed <1,500 (very low birth weight (VLBW)) or <2,500 grams (low birth weight (LBW), respectively (Figure 3A)). The percentages were similar for male and female neonates (16 and 24% for boys, and 18 and 29% for girls, respectively). The prevalence of VLBW was not affected by parity (20-30%; Figure 3C,D), but a birth weight between 1,500 and 2,500 grams was least frequent in male offspring of parity-2 mothers (10%; Figure 3C) and in female offspring of parity-3 and -4 mothers (~15%; Figure 3D). There were no neonates with a birthweight <2,000 gram in mothers with ≤2 previous offspring, but if the neonate was born at 40 weeks from subsequent pregnancies, such neonates appeared (Figure 2 (encircled)). These <2,000 gram newborns represented ~23% of the newborns of that age group, were born at ~60% of the weight of the corresponding non-affected group, were seen with a similar frequency in male and female newborns, and were only seen from the fourth offspring onwards in male neonates and from the fifth in female neonates (not shown). The percentage of male neonates in the cohort was at 45% significantly lower than that of female neonates (Table 1; P < 0.05), and dropped from ~50% during the first 3 completed pregnancies to only ~20% in mothers with 6 completed pregnancies (Figure 3B).

**Table 1:** **Total number of newborns with or without malformations in both hospitals**

Deliveries in six months	Total number %		Without malformation number %		With malformation number %	
Ayder referral hospital	503**	33	484	32	19	3.8
Mekelle hospital	1013**	67	1000	66	13	1.3
Males	684	45	662*	44	22*	3.2
Females	832	55	822	54	10	1.2
LBW	822	54	800	97	22	2.7
LBW Male	374	25	362	97	12	3.2
LBW Female	448	30	438	98	10	2.2
VLBW	530	35	515	97	15	2.8
VLBW Male	243	16	238	98	5	2.1
VLBW Female	287	19	277	97	10	3.5
Total deliveries	1516	100	1484	98	32	2.1

Percentages were calculated by taking the corresponding “total number” as denumerator.

Significance (*: P <0.05; **: P <0.001) was calculated between hospitals or between males and females.

### Spectrum of congenital anomalies

**Figure 4:**
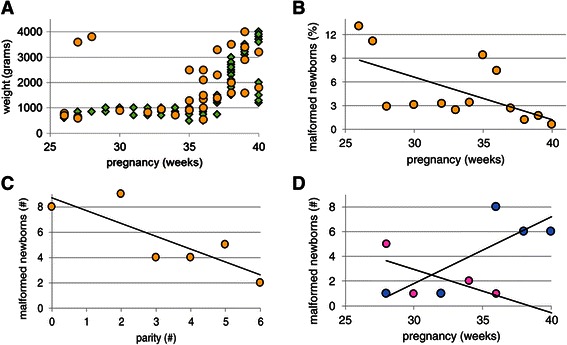
**Characteristics of newborns with an overt malformation. Panel A** shows the birth weight of offspring with an overt malformation (yellow symbols) superimposed on that of the non-malformed population (green symbols). The risk of having a malformed newborn decreases with increasing duration of the pregnancy (**panel B**; R^2^ = 0.38) and the number of deliveries (**panel C**; R^2^ = 0.68). **Panel D** shows that all malformed females (pink symbols) were born prior to 36 weeks of gestation (R^2^ = 0.45), whereas 90% of malformed males (blue symbols) were born after 36 weeks of gestation (R^2^ = 0.66).

The reported differences between male and female neonates in mothers with multiple pregnancies were reflected in the number of malformations. Among all neonates, only 32 (~2%) had visible congenital anomalies. The gestational age at which these babies were born is shown in Figure 4A. The chance of having a malformed baby declined after 35 weeks (Figure 4B; Table 2; P = 0.003). The number of parities (Figure 4C) and, to a lesser extent, the number gravidities also played a significant factor in determining the risk for neonatal anomalies (P < 0.001 and =0.061, respectively). Of the visibly malformed neonates, 10 were girls and 22 boys (Table 1; P = 0.044 (*χ*^2^ test)). The female newborns were all born before 36 weeks, whereas the 20 out of the 22 male newborns (~90%) were all born after 36 weeks gestation (Figure 4D). Male gender and low birthweight also increased the chance of having malformations (Table 2; P = 0.024 and 0.001, respectively). The results further showed that mothers with antenatal care had a slightly lower likelihood of having malformed neonates (Table 2; P = 0.037).

**Table 2:** **Logistic regression analysis of factors associated with the observed malformations**

Determinants	Coeff	SE	z	P value	95% CI
Gestational age	-0.266	0.090	-2.96	0.003	-0.441 - -0.089
Maternal age	0.096	0.095	1.01	0.311	-0.090 - +0.282
Gravidity	0.620	0.330	1.87	0.061	-0.029 - +1.269
Parity	1.496	0.253	5.90	<0.001	+0.999 - +1.992
Abortion history	-1.809	1.189	-1.52	0.128	-4.139 - +0.520
Antenatal care	-2.125	1.021	-2.08	0.037	-4.127 - -0.124
Maternal illnes	-4.894	2044.38	-0.00	0.998	-4012 - +4002
Medication	-7.614	962.38	-0.01	0.994	-1894 - +1879
Previous malformations	13.822	1803.71	0.01	0.994	-3521 - +3549
Sex	1.858	0.823	2.26	0.024	+0.243 - +3.472
Birth weight	-0.002	0.001	-3.29	0.001	-0.004 - -0.001

Table 3 shows that central nervous system (CNS) defects (spina bifida, hydrocephalus, meningocele, anencephaly) were present in 22 children (1.5% of all newborns), whereas anomalies in the musculoskeletal system (cleft lip, club foot, polydactyly) were present in 4. The remaining diagnosed malformations were present in the gastrointestinal system (n = 3; imperforate anus, abdominal wall defects) and genitourinary system (n = 1; hypospadia). In addition, 2 conjoined twins (parapagus and dicephalus) were found.

**Table 3:** **Congenital malformations according to anatomical systems**

Anatomical system	Number	% of all births
**CNS**	**22**	**1.45**
anencephaly	1	0.07
hydrocephalus	2	0.13
meningocele	1	0.07
spina bifida	18	1.18
**MSS**	**4**	**0.26**
cleft lip	1	0.07
club foot	1	0.07
polydactyly	2	0.13
**GUS**	**1**	**0.07**
hypospadia	1	0.07
**GIT**	**3**	**0.197**
imperforate anus	1	0.07
abdominal wall defect	2	0.13
**Others**	**2**	**0.13**
parapagus	1	0.07
dicephalus	1	0.07
**Total**	**32**	**2.11**

Note that these malformations were registered after inspection of the neonate only.

Table 4 shows that the babies with congenital anomalies died intrauterinely in 12 cases (0.8% of all births), during delivery in 7 cases (0.5% of all births), and within 24 hours after birth in 2 cases (0.1% of all births), while 11 infants with malformations (0.7% of all births) survived for >1 week. Males were overrepresented in all groups, but most prominently in the group with intrauterine death.

**Table 4:** **Status of the newborn with anomalies**

Death	Number	% of all births
Prenatal	12	0.79
Intra-partum	7	0.46
Post-partum	2	0.13
Alive	11	0.73
Total	32	2.11

## Discussion

The present study of a 6-months cohort of newborns in Northern Ethiopia reveals that newborns hardly increased in birth weight if born between 26 and 36 weeks, but rapidly thereafter. Thirty-five % of the cohort had a birth weight of <1,500 grams and 54% a birth weight of <2,500 grams. The prevalence of diagnosed malformations was 2.1%, but difficult to judge as only externally visible malformations were included. The number of CNS malformations was, at 1.5% of all births, very high. Males appeared to be more affected by the prevailing conditions in Northern Ethiopia than females: the offspring sex ratio was lower than reported elsewhere (~45% males) and declined dramatically to only ~20% males if the neonates were born to mothers with at least 3 completed pregnancies, which, as far as we are aware, was not reported earlier. Furthermore, malformations were more often seen in males than females (~60%). We estimated that ~25% of the pregnant women from the region of Mekelle and Enderta district had their delivery supervised in one of both hospitals, which leaves ample room for selection. One indication for selection is the higher prevalence of malformations in Ayder referral hospital than in Mekelle hospital. Antenatal care in Northern Ethiopia does, on the other hand, not yet include advanced diagnostic tools, such as an ultrasound examination. For this reason, we do not think that the population of women who delivered in the hospital was strongly biased by selection. The indices, therefore, show that the study reports an underpriviledged population, even by regional standards, and provides intriguing clues about the characteristics of the population, the risk factors that may lead to fetal congenital anomalies, and the pattern and occurrence of malformations in Northern Ethiopia. Thus far, this kind of study was nonexistent in Ethiopia, even though the death rate from congenital anomalies is believed to be high [17].

The birth weight of the sample population hardly increased between 26 and 36 weeks gestation and increased only thereafter. This relation between birth weight and increasing gestational age at birth is exceptional and best resembled the lowest percentile of the reference curves of neonates in a Western country, such as The Netherlands [18]. However, the latter curves show that a near absent increase in birth weight with increasing gestational age ended at 30-32 weeks, whereas we observed a near-absent increase in birth weight up to 36 weeks. Furthermore, we observed an prevalence of 35% VLBW and 54% LBW newborns. The percentage of LBW infants is almost 3-fold higher than that reported for Ethiopia as a whole (~20% LBW neonates [19]) or for the nearby region of Gondar, North-West Ethiopia (~17% LBW newborns [20]). The percentage of LBW newborns in Harare, Zimbabwe, was ~11% [21] and that in rural Gambia ~20% [8]. The percentage of VLBW infants is even more extreme, as the reported percentage of VLBW newborns in Kenya was only ~2% [22]. A comparison with the USA underscores the severity of the data: the percentage of LBW and VLBW newborns was ~13 and ~3, respectively, for non-Hispanic Blacks and 7-8 and 1.2, respectively, for the rest of the population [23], that is, the Ethiopian data are 4- and 12-fold as high as the most adversely disposed group in the USA. In our study, male offspring was best off if carried by a parity-2 mother and female offspring by a parity-2-4 mother. This trend fits with world-wide observations as far as nulli- and primiparae are concerned, but the relatively steep increase of the incidence of LBW does not [24]. Seasonal differences in food availability may account for up to ~2-fold difference in birth weight in some African countries [25]. In this respect, it should be mentioned that Northern Ethiopia has suffered socio-economically and nutritionally from war and repeated droughts in the past 2 decades. The findings suggest that many neonates in Northern Ethiopia are at risk, because the mortality in (V)LBW neonates is much higher than in normal-weight neonates [22,26,27].

The very high prevalence of severely underweight newborns after 3-4 prior deliveries (~60%) in our cohort is also noticeable. For Harare, Zimbabwe, a prevalence of ~37% of small-for-gestational-age (SGA) infants was reported [21]. Elsewhere, the appearance of SGA neonates is reportedly associated mainly with nutritional status [28], but not with parity [24]. In our cohort, LBW newborns, who were probably SGA, appeared concomitantly with a steep decline in the percentage of boys born in these pregnancies from ~50% in parity-0-3 women to only ~20% in parity-6 women. A low male-female sex ratio reportedly correlates with economic and environmental stress [29], parental age [29-31] or grand multiparity (≥5 births) [32]. However, our study population was young and grand multiparas represented only ~1% of the women. The human sex ratio at conception is ≥56% [33] and at birth >51% in favor of boys [34]. The severe, but brief Dutch famine at the end of World War II caused the sex ratio to decline to 42% [28], while parental age above 40 decreased the sex ratio even to ~35% [35]. Since the babies in the Dutch famine study were conceived prior to the famine, these data seem to show that the male genotype is less fit to deal with adverse conditions already prenatally. This sensitivity to adverse conditions is also reflected in the significantly higher prevalence of malformations in males.

The incidence of malformations was ~3.8% in Ayder referral hospital and 1.3% in Mekelle hospital. This result probably underestimates the actual percentage, because some of the major anomalies, such as cardiac defects with a prevalence of 12 per 1,000 live births in the Western world [36], were not observed. In agreement, a study in Uganda [14] did observe a relatively high prevalence of ~3% cardiovascular malformations (if minor anomalies like birth marks are excluded) [14]. In all likelihood, therefore, the percentage malformations in Northern Ethiopia is 5-10%, in agreement with a reported 1-5% of live born infants in Africa [11,37,38] and ~3-7% worldwide [39]. Consanguinity, a common cause of congenital malformations [40], is not common in the study area.

The most frequently observed malformations concerned the central nervous system (1.5% of all births and ~70% of all observed malformations), with the majority being spina bifida. This is probably due to the very visible presentation of these defects and, therefore, probably a parameter for less obvious malformations as well. In a similar study in Cameroon, 0.2% of the neonates suffered from CNS defects, of which spina bifida was the most prevalent with 73% [41]. In contrast, the prevalence of spina bifida among non-white Americans was only 0.04% and 3-fold lower than among white Americans [42]. In Uganda, spina bifida was hardly seen (0.13% of total births) [14,43,44]. These comparisons show how prevalent CNS malformations were in Northern Ethiopia. We were, of course, not able to determine the causes of the observed anomalies. However, some of the differences with other studies are certainly due to ethnic differences and the environment [45-48]. The relatively high incidence of spina bifida in our study (1.2% of all births) may relate folate deficiency, as dietary supplementation with folic acid around the time of conception strongly reduces the risk of spinal bifida in the offspring [49,50]. However, the very low and low birth weights of more than one third and more than half of the newborns make it likely that poor nutritional conditions in general play a more important role. Malnutrition (based on BMI; 2005) is present in 37.5% of adult females in Tigray and anemia in ~30%, while dietary energy consumption is below the minimum FAO approved level for ~40% of the population [19,51]. Since poor nutrition of the mother does affect the future capabilities and health of the offspring [52], intervention studies with the present study as reference are indicated and promising with respect to the identification of critical nutrients.

## Conclusion

The population of newborns with VLBW and LBW in Northern Ethiopia was high in 2012. Likewise, the percentage of newborns with CNS malformations was high. Male newborns appear more sensitive to the adverse conditions in Northern Ethiopia than female newborns.
